# A Single-Clamp Inchworm Actuator with Two Piezoelectric Stacks

**DOI:** 10.3390/mi15060718

**Published:** 2024-05-29

**Authors:** Lu Liu, Zheyang Ji, Yue Zhang, Huan Chen, Weimin Lou, Ming Kong

**Affiliations:** 1 College of Metrology and Measurement Engineering, China Jiliang University, Hangzhou 310018, China; lu_liu@cjlu.edu.cn (L.L.); jizheyang1123@163.com (Z.J.); zhangyue199909@gmail.com (Y.Z.); 2 Zhejiang Institute of Metrology, Hangzhou 310018, China; chenhuan_zjim@163.com (H.C.); lwmoptics@zju.edu.cn (W.L.)

**Keywords:** inchworm, piezoelectric actuator, single clamp, piezoelectric stack

## Abstract

Inchworm piezoelectric actuators have attracted much attention in the field of precision positioning due to the advantages of a large stroke, high output force, and high resolution. However, traditional inchworm piezoelectric actuators use two sets of clamps and a set of drive structures to achieve stepping motion, which generally requires at least three piezoelectric stacks, resulting in a complex structure and the control system. Several methodologies have been advanced to minimize the utilization of piezoelectric stacks. However, there still exists the issue of excessive volume. Therefore, an inchworm piezoelectric actuator with a single-clamp and single drive structure is proposed in the study, which provides a compact size and smaller volume. The clamping mechanism comprises two sets of clamping feet with opposite displacement, which alternate contact with the guide frame and adjustable plate to ensure that the clamping mechanism always has frictional force and accomplishes the stepping motion. The testing of the actuator’s step distance, output force, and other parameters was conducted utilizing a displacement sensor. Experimental results indicate that the actuator achieved a maximum speed of 174.3 μm/s and an output force of 8.6 N when the frequency and voltage were 19 Hz and 150 V.

## 1. Introduction

Piezoelectric actuators can directly convert electrical energy into mechanical energy. With its advantages of fast response time, large output force, and high accuracy, they are widely used in microrobotics [1], aerospace [2], precision machinery [3], electric lenses [4], precision inspection [5], etc. According to the working principle, piezoelectric actuators are mainly divided into ultrasonic [6,7,8,9], inertial drive [10,11], inchworm [12,13], and direct-push [14,15,16] types. The inchworm piezoelectric actuators are based on the motions of inchworms in nature, which accumulates the single-step displacement of the piezoelectric stacks, to achieve theoretically infinite displacement distance with nanoscale resolution and offer advantages such as high output force and high resolution compared to other types [17].

Most inchworm piezoelectric actuators have three main components: two clamping mechanisms and a driving mechanism which utilize three or more piezoelectric stacks. For example, Mohammad et al. [18] designed a vertical-crawling inchworm piezoelectric actuator employing a clamping mechanism to ensure self-locking capability in the event of power loss. Its maximum speed is 5.4 mm/s and the output force is 8.8 N. The overall dimension of the platform is 115 mm × 100 mm × 57.25 mm. Dong et al. [19]. designed an inchworm piezoelectric actuator primarily employing leveraged amplification and a bridge mechanism, achieving a maximum speed of 0.72 mm/s and a maximum displacement of 11 mm. However, more mechanisms and piezoelectric stacks can complicate the structures and circuits, and may cause control and vibration issues.

As a result, some of the latest inchworm piezoelectric actuators have adopted fewer piezoelectric stacks or simpler structural designs. Wang et al. [20] proposed a bidirectional inchworm actuator driven by two piezoelectric stacks. Its maximum speed was 0.22 mm/s, and the output force was 1.2 N. However, backward motion existed within linear motion. Building upon Wang’s work, Ma et al. [21] improved the design of the new actuator by utilizing a unique double rhombic bending hinge to transform the clamping foot into both a clamping structure and an extension structure. Such configuration effectively reduced backward motion and increased the maximum speed (0.47 mm/s) and the output force (11.76 N).

Liu et al. [22] proposed an actuator for precision positioning based on piezoelectric stack actuation and electrorheological fluids. Its maximum speed is 14.8 mm/s and the output force is 42 N. Lu et al. [23] proposed the use of magnetorheological elastomers for the inchworm actuator powered by batteries. Its maximum speed is 1.25 mm/s, and the output force is 0.42 N. Wang et al. [24] designed an actuator consisting of a piezoelectric stack, a DC motor, two permanent magnets, and a photoelectric sensor. The actuator is driven by only one channel DC signal, achieving a maximum speed of 0.77 μm/s and a maximum output force of 3.4 N. However, in order to minimize the number of piezoelectric stacks used, such structures compromise on volume, output force, and maximum speed.

Compared to the traditional inchworm piezoelectric actuator with two clamping mechanisms, the inchworm piezoelectric actuator designed in this paper utilizes only a single clamping mechanism, thus requiring only two piezoelectric stacks to achieve the inchworm’s operational principle. At the same time, it inherits the advantages of the traditional inchworm piezoelectric actuator, such as high output force and high resolution.

## 2. Structures and Working Principle

### 2.1. Structures of the Actuator and Roles of Each Mechanism

**Figure 1:**
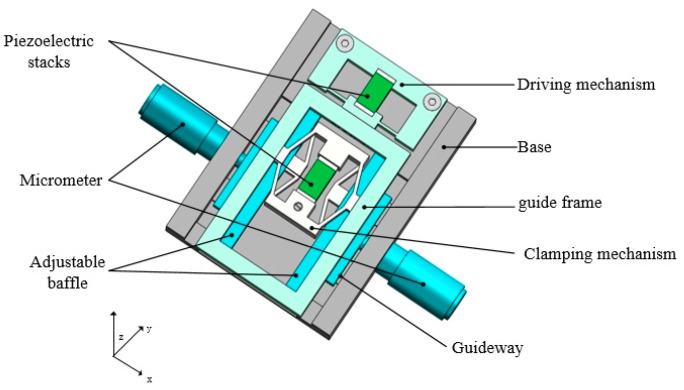
The model of the actuator.

**Figure 2:**
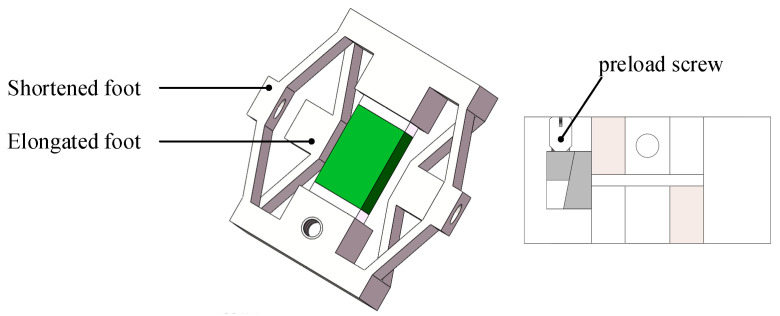
Structure of clamping mechanism.

The piezoelectric actuator proposed in this paper is composed of a driving mechanism, a clamping mechanism, and a guiding mechanism, as shown in Figure 1. The driving mechanism consists of a piezoelectric stack and an outer frame. The outer frame serves to secure the piezoelectric stack and extend its displacement. The clamping mechanism comprises two sets of triangular amplification structures with opposing displacements. This structure amplifies the output displacement of the piezoelectric stack and converts the displacement along the y-axis into displacement along the x-axis. The clamping mechanism includes a mortise and tenon structure and a set of preload screws, as shown in Figure 2. The mortise and tenon structure serves two purposes: first, it secures the piezoelectric stack tightly, ensuring that all its displacements can be amplified. Second, it adjusts the clamping force between the shortened foot and the guide frame.

The adjustable plate is mounted on the guide rails of the base and can move linearly on the base. With the help of two micrometers, the gap between the end of the elongated feet and the adjustable baffles can be quantitatively adjusted for precise contact.

Due to this design, the clamping mechanism remains in a clamped state. When no input voltage is applied to the clamping piezoelectric stack, the shortened foot of the clamping mechanism makes static contact with the guide frame, generating static friction, causing it to move in tandem with the driving mechanism. When a ramp voltage is applied to the clamping piezoelectric stack, the shortened foot disengages from the guide frame, while the elongated foot makes static contact with the adjustable plate, generating static friction. The overall size of this structure is 60 mm × 45 mm × 14 mm without the micrometer and can be detached from the laboratory.

### 2.2. Working Principle

**Figure 3:**
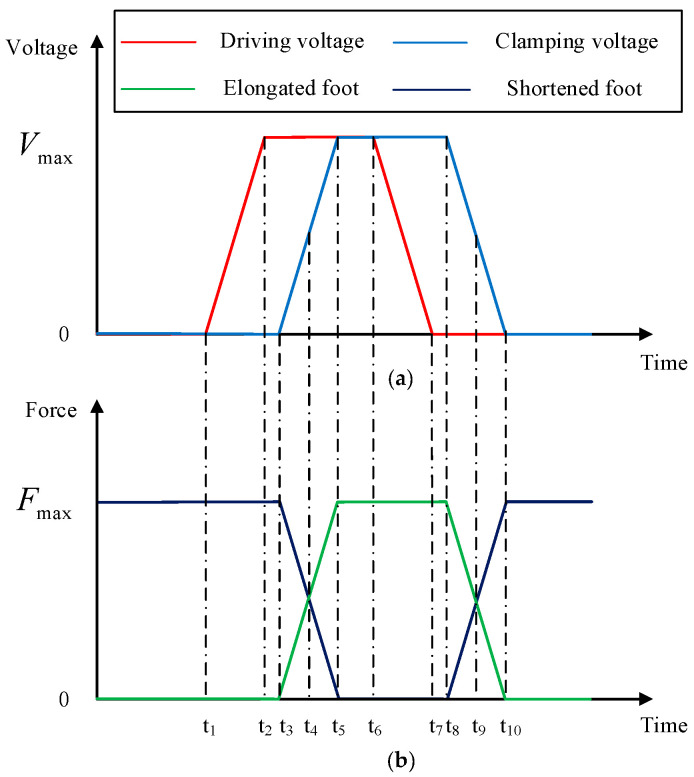
(**a**) Input voltage. (**b**) Clamping force.

**Figure 4:**
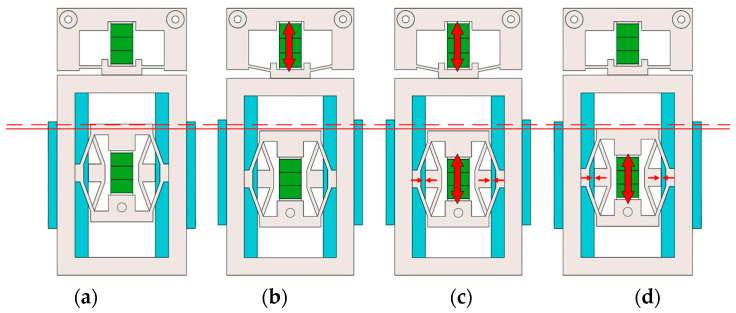
The working process of the proposed actuator of (**a**) Stage a; (**b**) Stage b; (**c**) Stage c; (**d**) Stage d.

Figure 3 shows the variation of voltage across two piezoelectric stacks of the actuator and the clamping forces of two sets of clamping feet at different stages over time. Figure 4 illustrates the different stages within one cycle of the actuator.

Step (0): from time 0 to $t_{1}$, it is the initial state of this piezoelectric actuator with no voltage input; the shortened foot is preloaded. The clamping force between the shortened foot and the guide frame is $F_{1}=F_{\max}$. The clamping force between the elongated foot and the guide frame is $F_{2}=0$. Therefore, the designed actuator features self-locking capability under power-off state, as shown in Figure 4a.

Step (1): from time $t_{1}$ to $t_{2}$, applying a ramped voltage to the driving piezoelectric stack, the driving piezoelectric stack extends and the driving mechanism generates a displacement corresponding to the elongation of the piezoelectric stack, propelling the guide frame forward with the clamping mechanism and maintaining the maximum voltage state until time $t_{6}$ as shown in Figure 4b.

Step (2): from time $t_{3}$ to $t_{5}$, applying a ramped voltage to the clamping piezoelectric stack, the clamping piezoelectric stack extends and the shortened foot of the clamping mechanism gradually shortens, disengaging from the guide frame. As the input voltages increase, the clamping force between the shortened foot and the guide frame reduces to 0. The elongated foot gradually extends and the clamping force between the elongated foot and the adjustable baffle reduces to $F_{\max}$. At time instant $t_{5}$, $F_{1}=0$, $F_{2}=F_{\max}$. This clamping force is maintained until time $t_{8}$, as shown in Figure 4c.

Step (3): from time $t_{6}$ to $t_{7}$, falling ramp voltages are applied on the driving piezoelectric stack. The driving mechanism, along with the guide frame, returns to the initial state, and the clamping mechanism remains in place, as shown in Figure 4d.

Step (4): from time $t_{8}$ to $t_{10}$, falling ramp voltages are applied on the clamping piezoelectric stack. The shortened foot of the clamping mechanism gradually extends and the clamping force between the shortened foot and the guide frame reduces to $F_{\max}$. The elongated foot gradually shortens and the clamping force between the elongated foot and the adjustable baffle reduces to 0. At time instant $t_{10}$, $F_{1}=F_{\max}$, $F_{2}=0$, returning to the initial state as shown in Figure 4a.

By analyzing the above-described operational process, in one cycle, the theoretical forward distance of the piezoelectric actuator is equal to the elongation displacement of the driving mechanism’s piezoelectric stack.

## 3. Results and Discussion

### 3.1. Mechanical Modeling and Analysis of Actuators

#### 3.1.1. Physical Analysis

**Figure 5:**
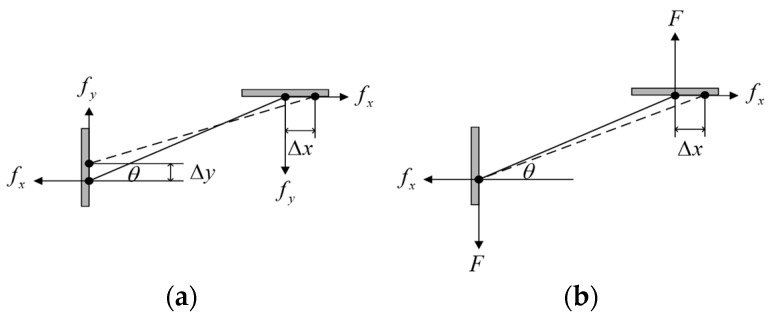
(**a**) The structure analysis of the shortened foot. (**b**) The structure analysis of the elongated foot.

During the motion process, the dual-layer triangular amplification mechanism of the clamping mechanism transforms the displacement of the piezoelectric stack into a clamping direction displacement. The parameters of the dual-layer triangular amplification mechanism directly affect the performance of the clamping mechanism. Therefore, it is necessary to analyze the performance of each individual triangular amplification mechanism before determining the parameters of the clamping mechanism. The output force of piezoelectric stack of the clamping mechanism is $F_{\mathrm{PZT}}$, and the preloading force on the shortened foot is $F_{N}$. Assuming that the elongated foot and the shortened foot do not affect each other, we consider the shortened foot as an example. When the piezoelectric stack elongates by Δx in the *x* direction, its shortened foot shortens by Δy in the *y* direction. Due to the symmetry of the actuator in the *x* and *y* directions, we can analyze only a quarter of the structure, as shown in Figure 5a, and the following relationship holds: $f_{x}=F_{\mathrm{PZT}}/4$; $f_{y}=F_{N}/4$. Due to the minimal change in angles, the rotation angles of points A and B are both denoted as θ. Point A moves in the *x* direction while point B moves in the *y* direction. Due to the constraints of the two points, the bridge arm beam is affected by the $2M_{r}$ moment. The following expression can be obtained according to the moment balance equation:

(1) $$M_{r}=\frac{f_{x}l\sin\theta +f_{y}l\cos\theta }{2}$$

Along the length of the bridge arm beam, AB is subjected to tensile force. In the direction perpendicular to the bridge arm beam, AB experiences a couple of forces. Additionally, the constraints at points A and B result in the presence of a moment force, denoted as $2M_{r}$, which causes the bending of the bridge arm beam during its motion. According to the equations of force balance and moment balance, the force and moment along the AB direction at distance *μ* from point A can be expressed as follows:

(2) $$\left\{\begin{array}{l} M=M_{r}-(f_{x}u\sin\theta +f_{y}u\cos\theta )\\ f_{u}=f_{x}\cos\theta -f_{y}\sin\theta \end{array}\right.$$

The total deformation energy $V_{\varepsilon }$ of the bridge arm beam is composed of tensile strain energy $V_{\varepsilon 1}$ and bending strain energy $V_{\varepsilon 2}$, which can be expressed as follows:

(3) $$V_{\varepsilon }=V_{\varepsilon 1}+V_{\varepsilon 2}$$

Based on Castigliano’s theorem, the displacement of the shortened foot in the *x* direction can be expressed as

(4) $$\Delta x_{1}=\frac{\partial V_{\varepsilon }}{\partial f_{x}}=\int_{0}^{l}\frac{f_{u}}{EA}\frac{\partial f_{u}}{\partial F_{pi}}dx=\frac{l}{EA}(f_{x}\cos\theta -f_{y}\sin\theta )\cos\theta$$

(5) $$\Delta x_{2}=\int_{0}^{l}\frac{M}{EA}\frac{\partial M}{\partial F_{pi}}dx=\frac{l^{3}}{12EI}(f_{x}\sin\theta +f_{y}\cos\theta )\sin\theta$$

(6) $$\Delta x=\Delta x_{1}+\Delta x_{2}=\frac{l}{EA}(f_{x}\cos\theta -f_{y}\sin\theta )\cos\theta +\frac{l^{3}}{12EI}(f_{x}\sin\theta +f_{y}\cos\theta )\sin\theta$$

Similarly, the displacement of the shortened foot in the *y* direction can be expressed as

(7) $$\Delta y=\Delta y_{1}+\Delta y_{2}=\frac{l}{EA}(f_{x}\cos\theta -f_{y}\sin\theta )\sin\theta +\frac{l^{3}}{12EI}(f_{x}\sin\theta +f_{y}\cos\theta )\cos\theta$$

The stiffness of the shortened foot in the *y* direction $K_{1}=2f_{y}/\Delta y$ can be calculated by Equation (7).

After analyzing the shortened foot, a similar analysis is conducted for the elongated foot, as shown in Figure 5b. Due to the adjustable plate, the displacement at point A equals 0, Δy=0. At the same time, in the initial state, there is no external force acting on it, $F_{N}=0$. However, in the working state, the elongated foot is subjected to force F and can obtain the following expression:

(8) $$f_{u}=f_{x}\cos\theta +F\sin\theta$$

Force can be obtained by substituting Δy=0 into Equation (7).

(9) $$F=\frac{(12I+Al^{2})f_{x}\sin\theta \cos\theta }{Al^{2}\cos^{2}\theta -12I\sin^{2}\theta }$$

Similarly, we can prove that

(10) $$\Delta x=\Delta x_{1}+\Delta x_{2}=\frac{l}{EA}(f_{x}\cos\theta +F\sin\theta )\cos\theta +\frac{l^{3}}{12EI}(f_{x}\sin\theta -F\cos\theta )\sin\theta$$

The stiffness of the elongated foot in the *y* direction $K_{2}=F/2\Delta y$ can be calculated by Equations (7) and (9). Equations (6), (7), (9) and (10) show that thickness h, length l, width a, and inclination angle θ affect the output displacement and the stiffness of the clamping mechanism.

#### 3.1.2. Parameter Selection

In the design process, the overall thickness of the clamping mechanism is set to 11 mm. Therefore, in the selection of parameters for the bridge arm beam, the focus is initially on the length, thickness, and inclination angle on the output performance of the clamping mechanism. A bridge arm beam with a thickness that is too small for the clamping mechanism can lead to inadequate stiffness, while an excessively large thickness can affect the displacement distance of the clamping foot. Similarly, an excessively small inclination angle for the bridge arm beam can result in insufficient rigidity of the clamping foot, while an excessively large inclination angle can affect the displacement distance of the clamping foot. Additionally, a bridge arm beam with a length that is excessively small in the clamping mechanism can affect the selection of piezoelectric stack, while a length that is too large can impact the overall size of the actuator.

As shown in Table 1, it was determined that the optimal magnification factor for the clamping mechanism ranges from 1.5 to 3. In accordance with the design specifications of the clamping mechanism and the performance characteristics of the piezoelectric stack, the optimal solution is determined to be l=7 mm, h=0.8 mm, θ=20° [25].

#### 3.1.3. Simulation Analysis

**Figure 6:**
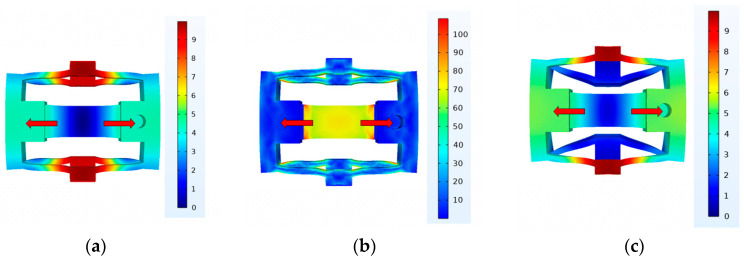

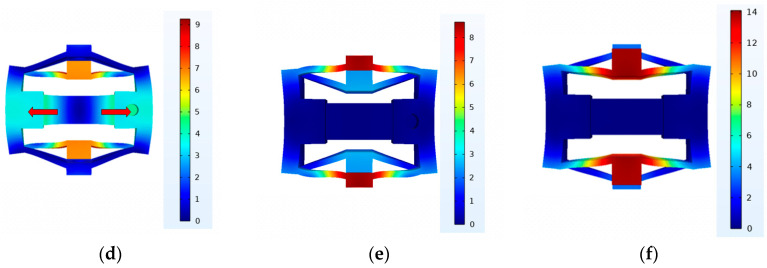
(**a**) Displacement of the clamping mechanism at 150 V (μm). (**b**) Stress of the clamping mechanism at 150 V (MPa). (**c**) A 70 N force is applied to the elongated foot while the piezoelectric stack is operating (μm). (**d**) A 100 N force is applied to the shortened foot while the piezoelectric stack is operating (μm). (**e**) A 100 N force is applied to the shortened foot while the piezoelectric stack is not operating (μm). (**f**) A 100 N force is applied to the elongated foot while the piezoelectric stack is not operating (μm).

Finally, based on the parameters determined in Section 3.1.2, we establish the clamping mechanism finite element model. The material of the proposed inchworm piezoelectric stack is set as spring steel 65 Mn; the density, Young’s modulus, and Poisson ratio are 7850 kg/m^3^, 1.98 × 10^11^ N/m^2^, and 0.282. When the clamping mechanism generates a 9.5 μm displacement along the direction of the piezoelectric stack, as shown in Figure 6a, the elongated foot exhibits a total displacement of 18.81 μm, while the shortened foot shows a total displacement of 18.19 μm. As shown in Figure 6b, the maximum stress in the clamping mechanism is 100 MPa, which is significantly below the yield strength of the chosen material, 65 Mn steel (430 MPa), meeting safety stress requirements. As shown in Figure 6c, when a 70 N force is applied to the elongated foot while the piezoelectric stack is operating, the displacement of the elongated foot is approximately 0 μm. As shown in Figure 6d, when a 100 N force is applied to the shortened foot while the piezoelectric stack is operating, the displacement of the shortened foot is approximately 0 μm. When the piezoelectric stack is not operational, applying a 100 N force on the shortened foot results in a displacement of approximately 16.31 μm for the shortened foot and about 2.73 μm for the elongated foot, as shown in Figure 6e. Similarly, when a 100 N force is applied to the elongated foot, the displacement of the elongated foot is approximately 25.86 μm, as shown in Figure 6f. Given the friction coefficient of 65 Mn steel as 0.17, the maximum clamping force in the initial state is calculated to be 37.92 N, and when the piezoelectric stacks are operational, the maximum clamping force is 20.34 N.

**Figure 7:**
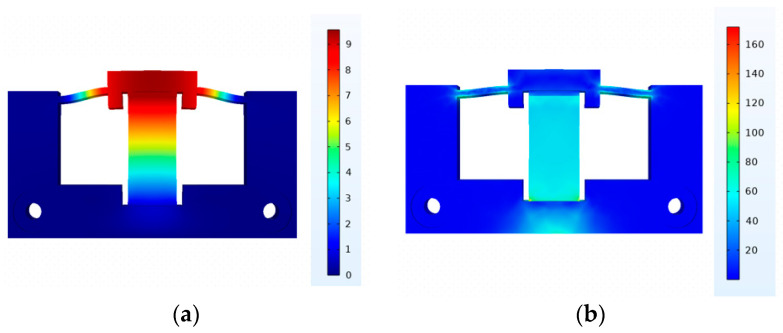
(**a**) Displacement of the driving mechanism at 150 V (μm). (**b**) Stress of the driving mechanism at 150 V (MPa).

Similarly, we establish the driving mechanism finite element model. Figure 7a illustrates the displacement of the driving mechanism in its operational state. As shown in Figure 7b, it is evident that the maximum stress is less than 180 Mpa, which is significantly below the yield strength of the chosen material for the actuation component, 65 Mn steel (430 Mpa), meeting safety stress requirements.

### 3.2. Experimental Evaluation

**Figure 8:**
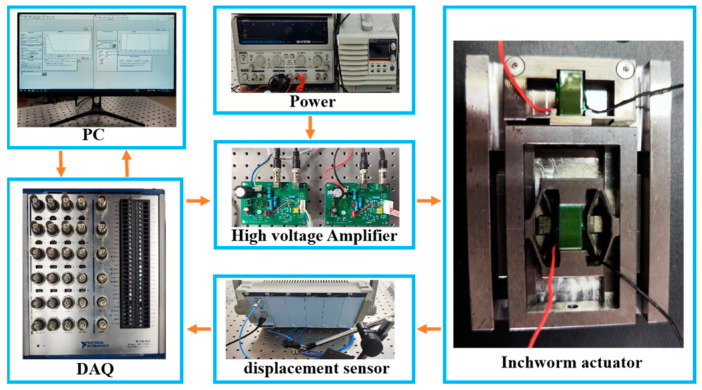
Experimental system.

The inchworm piezoelectric actuator is assembled, and an experimental system, as depicted in Figure 8, is constructed to investigate the performance of the proposed piezoelectric actuator designed. PC-driven data acquisition (DAQ) equipment (USB-6363, National Instruments, Austin, TX, USA) is applied to collect the position and provide excitation signals to the high voltage amplifier (magnification: 15×), which is used to drive the piezoelectric stack (Model: MTP150/5 × 5/10, CoreMorrow, Harbin, China. The displacement at 150 V is 9.5 μm ± 15%). This piezoelectric stack simultaneously satisfies the displacement requirement of the clamping mechanism while maintaining a small volume and high resolution. A capacitive displacement sensor (Model: E09.CAP200, CoreMorrow, China) with a measurement range of 200 μm and a measurement accuracy of 2.5 nm is employed to measure the output displacement of the actuator.

#### 3.2.1. Control Waveform Design

**Figure 9:**
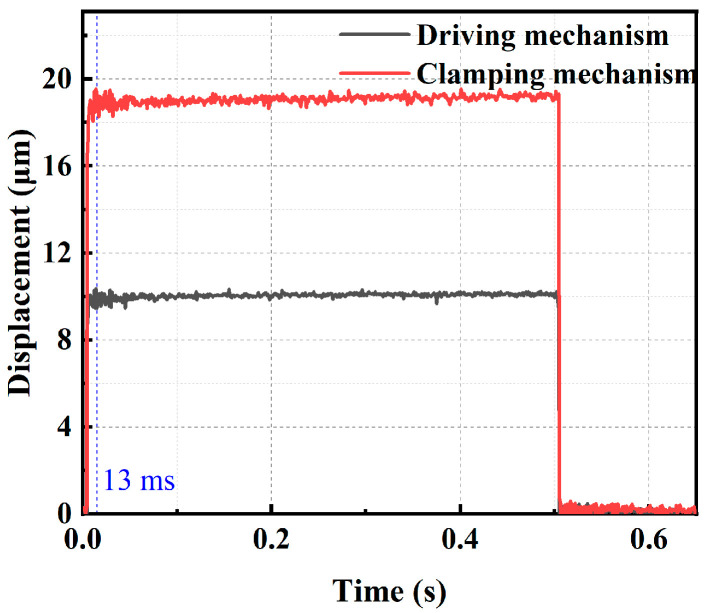
Response curves of the actuator.

The maximum operating speed of the piezoelectric actuator is constrained by the maximum frequency of the actuation signal. The maximum frequency of the actuation signal is limited due to various factors such as the bandwidth of the circuit, the resonant frequency of the mechanical structure, and the charging and discharging time of the piezoelectric stack. Here, the maximum operating speed of the actuator at a specific voltage is calculated by testing the output displacement response times of the driving and clamping mechanisms. Hence, a set of square wave signals (amplitude: 150 V, frequency: 1 Hz) is generated by the DAQ, and the collected output displacements of the two mechanisms are illustrated in Figure 9. The transition time for the output displacement curve of the driving and clamping mechanism from one steady state to another is 13 ms. Therefore, rise and fall times of the trapezoidal excitation signals are set as 13 ms. The highest operating frequency of the inchworm piezoelectric actuator is calculated to be 19 Hz.

#### 3.2.2. Output Characteristic Evaluation

**Figure 10:**
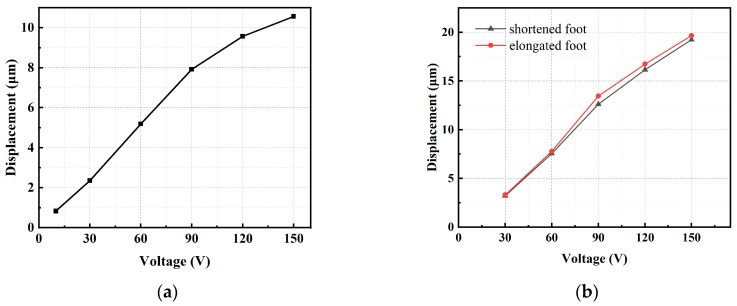
Displacements versus different voltages of (**a**) driving mechanism; (**b**) clamping mechanism.

The performance of the driving mechanism, illustrated in Figure 10a, directly influences the step size and speed of the actuator. It is essential to measure the relationship between the actuation distance and driving voltage. The output displacement of the actuator exhibits an approximately linear relationship with the driving voltage. When the driving voltage reaches 10 V, the output displacement of the driving mechanism is 0.83 μm. Similarly, when the driving voltage reaches 150 V, the output displacement of the driving mechanism increases to 10.56 μm. This indicates that the driving mechanism effectively harnesses the capability of the piezoelectric stack.

Based on the theoretical analysis, we can deduce that the two sets of clamping feet in the clamping mechanism impact the load capacity of the actuator. To ensure the stable operation of the actuator, it is necessary for the shortened foot to be completely disengaged from the push rod when the piezoelectric stack is energized, and the elongated foot should undergo a significant displacement when the piezoelectric stack is energized. Initially, it is essential to test the motion trajectories of the two sets of clamping feet in the clamping mechanism under no-load conditions. The assembled clamping mechanism is fixed, and a trapezoidal wave with a frequency of 1 Hz is supplied by a signal generator. The displacement of each clamping foot is measured using a capacitive displacement sensor. As depicted in Figure 10b, when the voltage reaches 150 V, the displacement of the shortened foot is 19.23 μm, and the displacement of the elongated foot is 19.65 μm.

**Figure 11:**
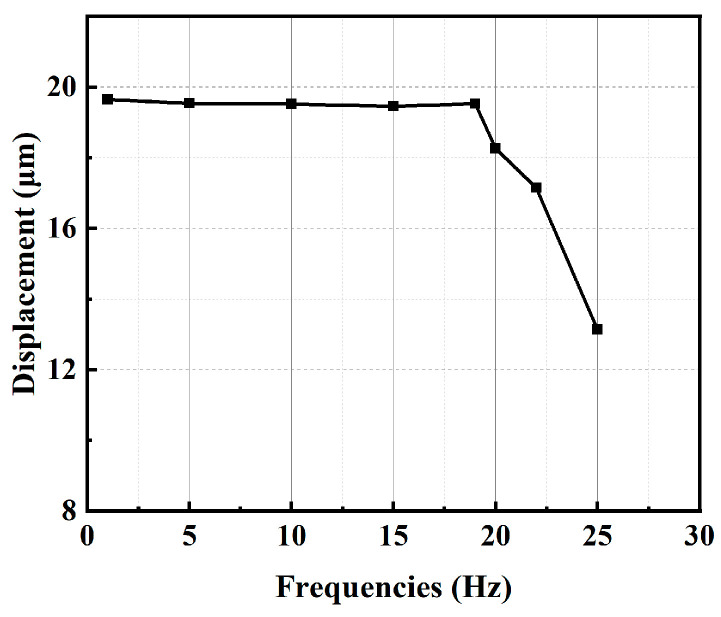
Displacements under different frequencies.

Examining how the displacement variation with changes in the driving signal frequency helps in understanding the effect of the piezoelectric stack’s capacitive characteristics on the output performance of the piezoelectric actuator. A square wave signal with voltage of 150 V, frequency of 1 Hz, and duty cycle of 50% is applied to the piezoelectric stack. By altering the signal frequency and measuring the output displacement amplitude at the side surface, a frequency response curve of the displacement amplitude is plotted, as shown in Figure 11.

#### 3.2.3. Output Characteristic Evaluation of the Inchworm Actuator

**Figure 12:**
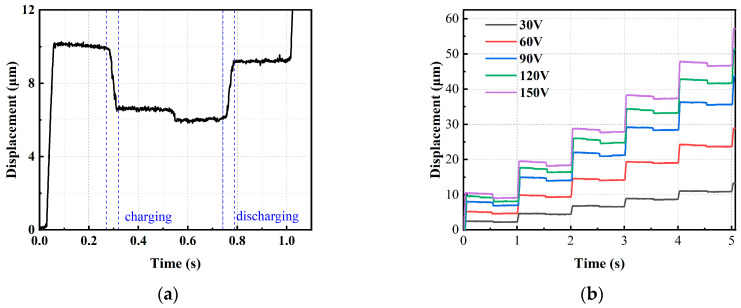

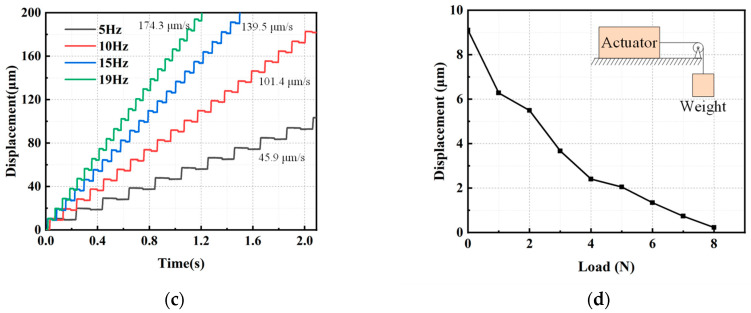
(**a**) Output displacement with clamping mechanism’s displacement. (**b**) Displacement outputs under different input voltages. (**c**) Displacement outputs under different input frequencies. (**d**) Loading capacity of the actuator.

A section of the output displacement is observed to be not smooth enough when measuring the output displacement at end of the clamping mechanism, as shown in Figure 12a. Through a thorough exploration of the actuator’s principles, we identified the causes of two instances of backlash. At 0.3 s, the displacement measurement point positioned at one end of the clamping mechanism resulted in an output displacement due to the operation of the piezoelectric stack within the clamping mechanism. Subsequently, when the piezoelectric stack discharged, it effectively subtracted this displacement, leading to insufficiently smooth output displacement. At 0.5 s, the transition of the clamping mechanism from a detached state to a contact state with the guide frame resulted in motion coupling. This occurred because of the face-to-face contact between the clamping mechanism and the guide frame, coupled with the limited machining precision, significantly exacerbating the issue of motion coupling. Therefore, a detection block was added in the middle of the clamping mechanism to eliminate the influence of the displacement of the clamping mechanism in the output displacement.

As depicted in Figure 12b, the displacement output of the proposed actuator is dependent on the driving voltages, with a frequency of 1 Hz for this particular case. The average step lengths are 9.4 μm, 8.4 μm, 7.2 μm, 4.7 μm, and 2.2 μm at driving voltages of 150 V, 120 V, 90 V, 60 V, and 30 V, respectively. When the driving voltage exceeds 30 V, the actuator exhibits a favorable stepping curve. During movement, a retraction motion of 1 μm occurs. This is attributed to the coupled motion generated by the clamping mechanism.

The forward curves for different frequencies within a 200 μm travel are illustrated in Figure 12c, demonstrating good consistency in step size. According to the working principle, the average output speed of the actuator increases with increasing frequency when the frequency is below 19 Hz. However, the actuator struggles to extend well when the frequency exceeds 19 Hz, resulting in a decrease in average speed. By calculation, the average speeds are 45.9 μm/s, 101.4 μm/s, 139.5 μm/s, and 174.3 μm/s at driving frequencies of 5 Hz, 10 Hz, 15 Hz, and 19 Hz, respectively.

Finally, to further investigate the output characteristics of the proposed actuator, the motion curves under different output forces were tested. The output force capability was tested by applying weights parallel to the slider, as shown in Figure 12d. The step distance decreases with the increase in output force, leading to a reduction in output speed. Under a voltage of 150 V and a frequency of 1 Hz, when the proposed actuator has output displacement, the maximum output force generated by the actuator can reach 8.6 N, demonstrating consistent load capacity for the proposed actuator.

### 3.3. Comparison

Based on the results measured above, the comparison of the basic performances among the proposed actuator and the other similar actuators are shown in Table 2. Compared to traditional piezoelectric actuators, the inchworm piezoelectric actuator designed in this paper utilizes only two piezoelectric stacks. However, actuators with only one piezoelectric stack result in larger volume and poorer performance. For actuators that use two piezoelectric stacks, the actuator designed in this paper has certain advantages in terms of volume and load capacity.

## 4. Conclusions

This paper presents a design of an inchworm actuator utilizing two piezoelectric stacks where the clamping mechanism employs two sets of clamping feet with opposite displacements, resulting in a simpler structure and control. The design undergoes detailed analysis and testing, providing a static model for clamping force and step size. Its effectiveness is validated through performance testing. Experimental results indicate that, with a 9% proportion of retraction motion, the maximum linear velocity is 174.3 μm/s, and the maximum output force is 8.6 N. This design, while reducing the number of piezoelectric stacks, retains the advantages of traditional linear displacement actuators, offering a compact size and the ability to operate outside laboratory environments.

## Figures and Tables

**Table 1:** Orthogonal experimental methodology.

l (mm)	h (mm)	θ (°)	Displacement of Elongated Foot (μm)	Displacement of Shortened Foot (μm)	Displacement under 100 N (μm)	Clamping Force (N)
7	1.0	25	15.9	16.8	38.0	88.4
7	0.8	15	20.4	21.4	72.0	59.4
7	0.6	20	22.9	22.5	148.0	30.4
6	1.0	15	16.8	18.9	28.6	132.1
6	0.8	20	20.0	20.0	46.0	86.9
6	0.6	25	19.4	19.3	96.0	40.2
5	1.0	20	15.9	16.9	16.0	211.3
5	0.8	25	16.9	17.0	28.0	121.4
5	0.6	15	25.7	25.7	66.8	76.9

**Table 2:** Comparisons between the values in this work and those of other similar actuators.

	Number of PSAs	Size (mm)	Speed (mm/s)	Output Force (N)
Mohammad et al. [18]	3	115 × 100 × 57.25	5.4	8.8
Dong et al. [19]	3	150 × 45 × 30	0.72	N/A
Wang et al. [20]	2	120 × 120 × 25	0.22	1.2
Ma et al. [21]	2	61 × 29 × 7	0.47	11.76
Lu et al. [23]	1	N/A	1.25	0.42
Wang et al. [24]	1	N/A	0.00077	3.4
This study	2	60 × 45 × 14	0.17	8.6

## Data Availability

The original contributions presented in the study are included in the article, further inquiries can be directed to the corresponding author.
